# Integration of Circulating Immune Checkpoint Proteins and Osteopontin Refined Risk Stratification in Osteosarcoma

**DOI:** 10.3390/cancers18111701

**Published:** 2026-05-23

**Authors:** Nguyen Tran Quang Sang, Nguyen Van Khanh, Hoang Hai, Tran Trung Dung, Tran Duc Thanh, Dang Minh Quang, Pham Tuan Anh, Nguyen Bui Tam Chi, Tran Van Bao, Nguyen Viet Trung, Tran Thi Thu Hien, Hidetomi Terai, Le Thi Thanh Thuy

**Affiliations:** 1 Department of Global Education and Medical Sciences, Graduate School of Medicine, Osaka Metropolitan University, Osaka 545-8585, Japan; v.sangntq@vinmec.com (N.T.Q.S.); v.khanhnv7@vinmec.com (N.V.K.); hoanghai@omu.ac.jp (H.H.); sg24401y@st.omu.ac.jp (P.T.A.); sx25284n@st.omu.ac.jp (N.B.T.C.); tvbao@huemed-univ.edu.vn (T.V.B.); 2 Orthopedic Trauma & Sports Medicine Center, Vinmec Times City International Hospital, Vinmec Healthcare System, Hanoi 100000, Vietnam; dungbacsy@dungbacsy.com (T.T.D.); v.thanhtd26@vinmec.com (T.D.T.); v.quangdm2@vinmec.com (D.M.Q.); 3 Department of Pathology, Vinmec Times City International Hospital, Vinmec Healthcare System, Hanoi 100000, Vietnam; v.trungnv42@vinmec.com; 4 Department of Orthopaedic Surgery, College of Health Science, VinUniversity, Hanoi 100000, Vietnam; 5 Department of Anesthesia and Pain Management, Vinmec Times City International Hospital, Vinmec Healthcare System, Hanoi 100000, Vietnam; v.hienttt@vinmec.com; 6 Department of Orthopaedic Surgery, Graduate School of Medicine, Osaka Metropolitan University, Osaka 545-8585, Japan; terai@omu.ac.jp

**Keywords:** osteosarcoma, immune checkpoint proteins, osteopontin, prognostic biomarkers, liquid biopsy

## Abstract

Background: Osteosarcoma remains the most common primary malignant bone tumor in children and adolescents, with largely unchanged survival outcomes in recent decades. Non-invasive biomarkers for preoperative risk stratification are urgently needed. Circulating immune checkpoint proteins (ICPs) and bone remodeling factors (BRFs) represent promising candidates; however, their combined prognostic value in osteosarcoma remains unclear. Methods: We prospectively analyzed plasma samples from 47 patients with osteosarcoma, selecting the earliest available sample before disease progression or the last follow-up. A panel of ICPs and BRFs was quantified using multiplex immunoassays. sHVEM and sCD27 were used to construct a two-marker ICP signature (ICP2); subtypes were defined by unsupervised consensus clustering (k = 2) applied to log_2_-transformed sHVEM and sCD27 concentrations, and sOPN was included for integrative analysis. Survival associations were assessed using Kaplan–Meier analysis and Firth’s penalized Cox regression. The model performance was evaluated using Harrell’s C-index. Results: Two ICP2 subtypes were identified (type1, *n* = 26; type2, *n* = 21). ICP2-type2 was associated with significantly worse progression free survival (PFS) (*p* = 0.0045) and overall survival (OS) (*p* = 0.0024) and remained an independent predictor after adjusting clinicopathological factors. The ICP2 model demonstrated high discriminative performance (C-index 0.924 for PFS and 0.903 for OS). Internal validation by leave-one-out cross-validation (LOOCV), in which consensus clustering was re-derived at each fold, yielded corrected C-indices of 0.612 for PFS and 0.806 for OS (versus apparent estimates of 0.687 and 0.689, respectively), confirming acceptable-to-good discrimination after accounting for overfitting. Elevated sOPN was associated with poorer survival in the exploratory analyses, although its prognostic value was less consistent under a pre-specified cutoff. In the combined models, sOPN provided modest additional value for OS stratification, with the grdgfdgICP2-type2/OPN-high subgroup exhibiting the poorest outcome. Conclusions: The circulating sHVEM/sCD27 signature defines prognostically distinct osteosarcoma subtypes with a strong discriminative performance. This minimally invasive model may support preoperative risk stratification. Integration with sOPN suggests a potential biological link between immune regulation and bone remodeling, warranting validation in larger cohorts.

## Simple Summary

Osteosarcoma is the most common bone cancer in children and adolescents, yet survival rates have barely improved over the past four decades. Doctors currently rely on information gathered during or after surgery to assess how serious a patient’s disease is, leaving limited opportunity to adapt treatment early. This study investigated whether proteins circulating in the blood-pecifically, immune-related proteins and a bone-derived protein called soluble osteopontin (sOPN)-could identify high-risk patients before surgery. By analyzing blood samples from 47 patients, the researchers found that two immune proteins, soluble Herpesvirus Entry Mediator (sHVEM) and soluble Cluster of Differentiation 27 (sCD27), could reliably classify patients into groups with markedly different survival outcomes. Combining these immune markers with sOPN further refined risk prediction. These findings suggest that a simple blood test measuring just three proteins may help clinicians identify osteosarcoma patients who need more aggressive treatment, potentially improving personalized care for this devastating disease.

## 1. Introduction

Osteosarcoma is the most common primary malignant bone tumor, predominantly affecting children and adolescents, with a peak incidence in the second decade of life [1,2,3]. Despite multimodal treatments, including neoadjuvant chemotherapy, surgical resection, and adjuvant chemotherapy, long-term outcomes have remained largely unchanged over the past four decades. The five-year OS rate is approximately 60–70% for patients with localized disease, which decreases to less than 20–30% in those with metastatic or recurrent disease [4,5,6]. Thus, there is an urgent need for improved prognostic tools and novel therapeutic strategies.

Current risk stratification for osteosarcoma relies primarily on clinicopathological features such as tumor size, histological response to neoadjuvant chemotherapy, surgical margins, and the presence of metastasis [7]. However, these parameters are typically available only during or after surgery, limiting their value for early treatment adaptation. In contrast, circulating biomarkers measurable before or during treatment could provide a minimally invasive means of identifying high-risk patients, enabling risk-adapted therapeutic strategies and more precise clinical decision-making [8,9]. Recent studies investigating soluble immune checkpoint molecules have highlighted their potential to open new avenues of research in osteosarcoma. Binghao Li and colleagues reported that elevated levels of sTIM3, sCD137, sIDO, and sCTLA4 were significantly associated with osteosarcoma risk (all *p* < 0.05) [8]. In addition, sBTLA, sPDL2, and sCD27 were significantly correlated with the risk of lung metastasis, while increased sBTLA and sTIM3 levels were associated with disease progression [8]. Furthermore, Hanqi Peng and colleagues demonstrated that patients who developed metastasis exhibited elevated circulating levels of CD48, B7-H2, TIMD-4, B7-H6, CD134, B7-H5, CD47, and S100A8/A9, all of which were associated with a higher metastatic risk [10]. Together, these findings suggest that circulating immune checkpoint-related proteins may serve as promising minimally invasive biomarkers for prognostic stratification and metastatic prediction in osteosarcoma.

The tumor immune microenvironment has emerged as a key determinant of osteosarcoma progression. Tumor cells can evade immune surveillance through the expression of inhibitory immune checkpoint molecules, and the presence of tumor-infiltrating lymphocytes has been associated with improved clinical outcomes [11,12]. Soluble forms of ICPs generated through proteolytic shedding or alternative splicing are detectable in peripheral blood and may reflect systemic immune activity [10,13]. HVEM and CD27 are members of the tumor necrosis factor receptor superfamily that play central roles in T-cell activation and immune regulation [14]. Although dysregulation of these pathways has been reported in multiple malignancies, their prognostic relevance as circulating biomarkers of osteosarcoma remains poorly understood [8,15,16].

Osteosarcoma arises within the bone microenvironment, where dynamic interactions between osteoblasts and osteoclasts regulate bone remodeling. Disruption of this balance contributes to tumor progression and metastasis. sOPN, a multifunctional secreted glycoprotein involved in bone matrix remodeling, cell adhesion, and immune modulation, has been implicated in tumor progression and metastatic dissemination in various cancers [17,18,19]. Whether bone remodeling factors, such as OPN, can complement immune-based biomarkers to improve risk stratification in osteosarcoma has not yet been systematically investigated.

Taken together, these observations suggest a potential interaction between systemic immune regulation and bone remodeling in osteosarcoma progression. We hypothesized that circulating ICPs define biologically and clinically distinct osteosarcoma subtypes, and that integration with bone remodeling factors may further refine prognostic stratification. To test this hypothesis, we evaluated a panel of circulating ICPs and BRFs in plasma samples from patients with osteosarcoma and assessed their association with clinical outcomes.

## 2. Materials and Methods

### 2.1. Study Design and Participants

This prospective cohort study enrolled 56 patients with pathologically confirmed osteosarcoma treated at the Vinmec Healthcare System, Hanoi, Vietnam, between 2021 and 2023. Of these, 47 patients met the eligibility criteria and were included in the final analysis. Eligible patients had (i) histologically confirmed osteosarcoma, (ii) availability of at least one plasma sample collected prior to any documented disease progression, and (iii) adequate clinical follow-up data for survival analysis. Patients with a prior malignancy, active autoimmune disease, or recent systemic immunosuppressive therapy were excluded.

For biomarker analysis, plasma samples were collected at two pre-specified time points: baseline prior to initiation of neoadjuvant chemotherapy (*n* = 12) and immediately before surgical resection following completion of neoadjuvant chemotherapy (pre-surgery; *n* = 35). The identified circulating biomarker signature primarily reflects the preoperative immune landscape after neoadjuvant chemotherapy. One plasma sample per patient was used, which was defined as the earliest available sample collected prior to disease progression. Patients were classified based on subsequent clinical outcomes during follow-up, while biomarker measurements were obtained prior to these events. The non-advanced group (*n* = 34) comprised patients who remained progression-free, whereas the advanced group (*n* = 13) included patients who developed disease progression, defined as local recurrence, distant metastasis, or death.

Demographic and clinicopathological variables included age, sex, body mass index (BMI), tumor location, tumor size, histological subtype, Enneking stage, serum alkaline phosphatase (ALP), and lactate dehydrogenase (LDH). Huvos histological response grading was performed in patients receiving neoadjuvant chemotherapy.

PFS was defined as the time from diagnosis to recurrence, metastasis, or death. OS was defined as the time from diagnosis to death. Patients without adverse events were censored at the last follow-up visit. The median follow-up time was 23.7 months (95% Confidence Interval (CI): 21.4–27.3; range: 4.6–50.6), as estimated by the reverse Kaplan–Meier method. No formal sample size calculation was performed given the exploratory nature of the study.

### 2.2. Plasma Sample Processing and Multiplex Immunoassay

Peripheral blood samples were collected into Ethylenediaminetetraacetic acid (EDTA) tubes and processed within 2 h. Plasma was separated by centrifugation at 2000× *g* for 15 min at 4 °C using a single centrifugation step without platelet depletion. Plasma aliquots were stored at −80 °C at the collecting institution in Vietnam until shipment on dry ice to Osaka Metropolitan University (Japan) for centralized batch analysis. Prior to assay, samples were thawed, vortexed, and briefly centrifuged to remove particulates. All multiplex immunoassays were performed in a single analytical batch to minimize inter-batch variability.

Circulating ICPs and BRFs were measured using multiplexed fluorescent bead-based immunoassays on the Luminex Bio-Plex 200 system (Bio-Rad, Hercules, CA, USA) as previously described [20,21,22]. Soluble ICPs were measured using the MILLIPLEX^®^ MAP Human Immuno-Oncology Checkpoint Protein Panel 1 (Cat# HCKP1-11K-PX17, MilliporeSigma, Burlington, MA, USA), which simultaneously quantifies 17 analytes: sBTLA, sCD27, sCD28, sTIM-3, sHVEM, sCD40, sGITR, sLAG-3, sTLR-2, sGITRL, sPD-1, sCTLA-4, sCD80 (B7-1), sCD86 (B7-2), sPD-L1, sICOS, and sPD-L2. Circulating BRFs were measured using the MILLIPLEX^®^ MAP Human Bone Magnetic Bead Panel (HBNMAG-51K, MilliporeSigma), which quantifies 13 analytes: sclerostin, DKK-1, IL-1β, ACTH, IL-6, osteocalcin, leptin, Osteoprotegerin, insulin, parathyroid hormone, OPN, TNF-α, and FGF-23.

Both assays were performed according to the manufacturer’s overnight protocol with minor modifications. Plasma samples were diluted 1:2 in Assay Buffer and added (25 µL/well) to antibody-immobilized magnetic bead mixtures in a 96-well plate. Plates were incubated overnight (16–18 h) at 2–8 °C (ICPs panel) or 4 °C (BRFs panel) with continuous agitation (500–800 rpm), followed by magnetic washing 3 times with 200 µL Wash Buffer (Cat# L-WB, MilliporeSigma, Burlington, MA, USA). Detection Antibodies and Streptavidin-Phycoerythrin (SA-PE) were added sequentially per panel-specific protocols. Beads were resuspended in Sheath Fluid PLUS and acquired on the Luminex^®^ 200™ reader (minimum 50 beads per analyte region; gate settings: 8000–15,000; Reporter Gain: Default low PMT). Median fluorescent intensity (MFI) values were fitted to a 5-parameter logistic (5-PL) standard curve using MILLIPLEX^®^ Analyst 5.1. All samples were measured in duplicate, and mean values were used for downstream analyses. Assay performance was monitored using two kit-supplied quality control samples (QC1 and QC2) per plate. Manufacturer-validated intra-assay CVs were <5% and inter-assay CVs <10% for the ICPs panel, and <10% and <15%, respectively, for the BRFs panel.

### 2.3. Marker Selection and Subtype Definition

Candidate ICP markers were selected using a three-step approach: (i) differential expression between the advanced and non-advanced groups, (ii) association with PFS in univariate analysis, and (iii) biological relevance. sHVEM and sCD27 were prioritized based on consistent signals across differential expression (nominal significance), survival association, and biological relevance to construct an ICP2. Given the exploratory nature and limited sample size, no formal multiple testing correction was applied.

Unsupervised consensus clustering was performed using the ConsensusClusterPlus package (R) [23] on log_2_-transformed and scaled sHVEM and sCD27 concentrations. The clustering parameters included k = 2 and 1000 resampling iterations, using hierarchical clustering with Pearson correlation distance. Two subtypes were defined (ICP2-type1 and ICP2-type2).

Among the BRF analytes, sOPN was selected based on biological relevance and exploratory association with survival. sOPN showed a trend toward higher levels in advanced disease but did not reach statistical significance. For the integration analyses, sOPN was dichotomized at the cohort median (log_2_-transformed concentration) to minimize overfitting.

A combined variable was constructed by crossing the ICP2 subtype and sOPN levels, yielding four subgroups: ICP2-type1/OPN-low (T1-OL), ICP2-type1/OPN-high (T1-OH), ICP2-type2/OPN-low (T2-OL), and ICP2-type2/OPN-high (T2-OH). Discordant combinations (T1-OH and T2-OL) were defined as intermediate risk.

### 2.4. Statistical Analysis

Continuous variables were expressed as medians with interquartile ranges (IQR) and compared using the Mann–Whitney U test. Categorical variables were presented as counts and percentages and compared using Pearson’s χ^2^ test or Fisher’s exact test, as appropriate.

Kaplan–Meier survival curves were generated and compared using log-rank tests. For exploratory analyses of individual markers, optimal cutoff points were determined using maximally selected rank statistics (minprop = 0.20). sOPN was dichotomized using a pre-specified median cutoff for the integrated model.

Multivariate Cox regression was performed using Firth’s penalized likelihood (coxphf package, v1.13) [24] to account for the separation in small samples. The primary model (*n* = 47) was adjusted for age group, sex, and tumor location. Tumor size was excluded because of missing data and was included only in the sensitivity analyses (*n* = 32). Missing data was handled using complete-case analysis.

Model discrimination was evaluated using Harrell’s concordance index (C-index) derived from Cox proportional hazards models. Apparent model performance was first estimated using the entire cohort (*n* = 47).

To internally validate the prognostic performance of the ICP2 subtype and reduce the risk of overfitting associated with the limited sample size, LOOCV was applied.

Unsupervised consensus clustering was performed based on the expression levels of selected biomarkers. To visualize the clustering structure and assess the separation between subgroups and t-Distributed Stochastic Neighbor Embedding (t-SNE) were employed.

Logistic regression models were used to evaluate classification performance for advanced disease status (binary outcome). The variables included in the logistic regression models were age, sex, tumor location, ICP2 subtype, and sOPN levels. Receiver operating characteristic (ROC) curves and DeLong’s test were used to compare model discrimination.

All statistical analyses were performed using R (v4.4.1), including the survival, survminer, ConsensusClusterPlus, and coxphf packages. Two-sided *p* < 0.05 was considered statistically significant.

## 3. Results

### 3.1. Patient Characteristics

A total of 47 patients with pathologically confirmed osteosarcoma, comprising 34 non-advanced and 13 advanced cases, were included in the final biomarker analysis cohort (Table 1). Over the entire follow-up period, 13 PFS events (progression or death) and 10 OS events (death) were recorded, all occurring in the advanced group. The cohort comprised 29 male (61.7%) and 18 female patients. Median age was similar between groups (15.5 [IQR, 12.2–18.0] vs. 16.0 [12.0–18.0] years; *p* = 0.774). Most tumors were located in the extremities, with no significant difference between groups (non-extremity tumors: 8.8% vs. 15.4%; *p* = 0.902).

No statistically significant differences were observed between patients with non-advanced and advanced disease in terms of age, sex, BMI, tumor location, tumor size, histological subtype, or LDH levels (all *p* > 0.05). Most tumors in both groups were classified as Enneking stage IIB (94.1% vs. 100.0%; *p* = 1.000). Serum ALP levels were significantly higher in patients with advanced disease (median 194.9 vs. 106.4 U/L; *p* = 0.022). Among 26 evaluable patients receiving neoadjuvant chemotherapy, histological response (Huvos grade) did not differ significantly between groups (*p* = 0.716), nor did the proportion of patients achieve a good response (≥90% necrosis; 47.4% vs. 57.1%; *p* = 1.000).

As defined by the study criteria, all patients with advanced disease progression during the follow-up. Specifically, 12 of the 13 (92.3%) patients developed metastasis, and 7 of the 13 (53.8%) experienced recurrence, whereas no such events were observed in the non-advanced group (both *p* < 0.001). Across the entire cohort, 13 PFS events (27.7%) were recorded. Accordingly, PFS was significantly shorter in the advanced group (median 395.0 [IQR 320.0–454.0] vs. 680.0 [433.0–833.0] days; *p* = 0.003).

OS analysis was limited by the absence of events in the non-advanced group, precluding a reliable estimation of the median OS. A total of 10 OS events (deaths; 21.3%) were observed across the cohort, all occurring in the advanced group (76.9%). Although no statistically significant difference in OS was observed between the groups (*p* = 0.461), this finding should be interpreted with caution, given the imbalance in event rates (76.9% vs. 0%).

### 3.2. Soluble Immune Checkpoint Proteins Associated with Osteosarcoma Progression

**Figure 1:**
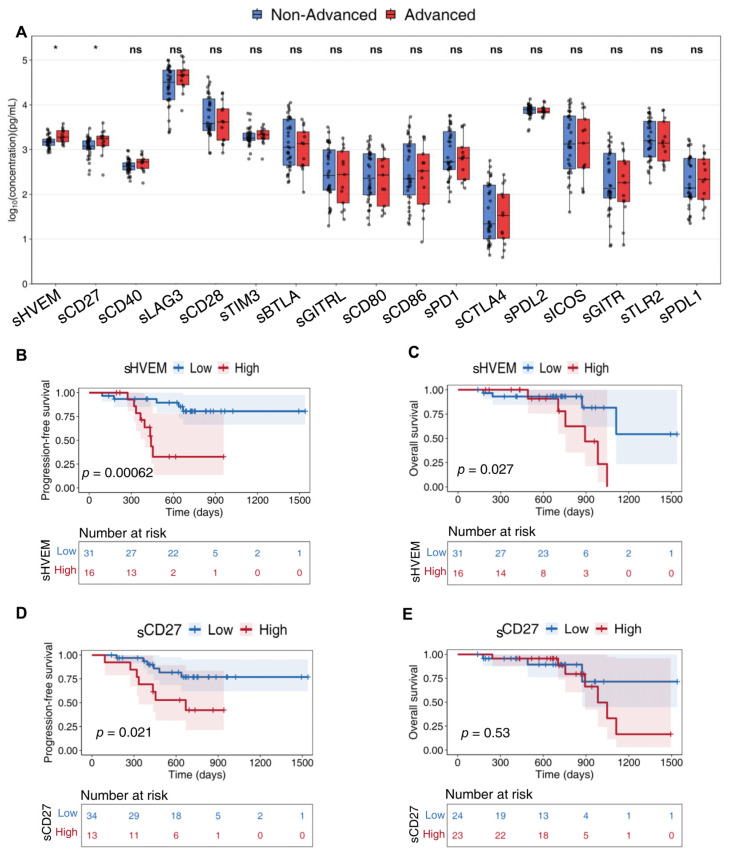
Soluble ICPs are associated with survival outcomes in osteosarcoma. (**A**) Box plots showing circulating concentrations of 17 soluble ICPs (log10 pg/mL) in non-advanced (blue) and advanced (red) patients. Individual data points are overlaid. *p*-values were calculated using the Mann–Whitney U test; * *p* < 0.05, ns = not significant. Only sHVEM and sCD27 showed nominal differences between groups. (**B**,**C**) Kaplan–Meier curves for PFS (**B**) and OS (**C**) stratified by sHVEM levels (Low, *n* = 31; High, *n* = 16). (**D**,**E**) Kaplan–Meier curves for PFS (**D**) and OS (**E**) stratified by sCD27 levels (Low, *n* = 34/24; High, *n* = 13/23 for PFS/OS, respectively).

Multiplex immunoassays were used to quantify 17 soluble ICPs in plasma samples from patients with non-advanced and advanced osteosarcoma. Among these analytes, sHVEM and sCD27 showed nominal differences between the groups (*p* = 0.022 and *p* = 0.031, respectively), whereas no other markers remained significant after correction for multiple comparisons (Figure 1A; Table S1). Logistic regression analysis did not identify any individual ICP as an independent predictor of advanced disease status, suggesting that a single analyte may be insufficient for capturing disease heterogeneity (Table S2).

To evaluate the prognostic relevance, Kaplan–Meier analyses were performed using data-driven cutoff points derived from maximally selected rank statistics. These analyses were considered exploratory. Elevated sHVEM levels were consistently associated with shorter PFS (*p* = 0.00062) and OS (*p* = 0.027) (Figure 1B,C). High sCD27 levels were associated with shorter PFS (*p* = 0.021), but not OS (*p* = 0.53), suggesting potential endpoint-specific effects (Figure 1D,E). Taken together, these findings support sHVEM and sCD27 as candidate biomarkers with complementary prognostic relevance for osteosarcoma.

### 3.3. Unsupervised Clustering of Circulating Immune Checkpoint Proteins Identifies Two Prognostically Distinct Subtypes

**Figure 2:**
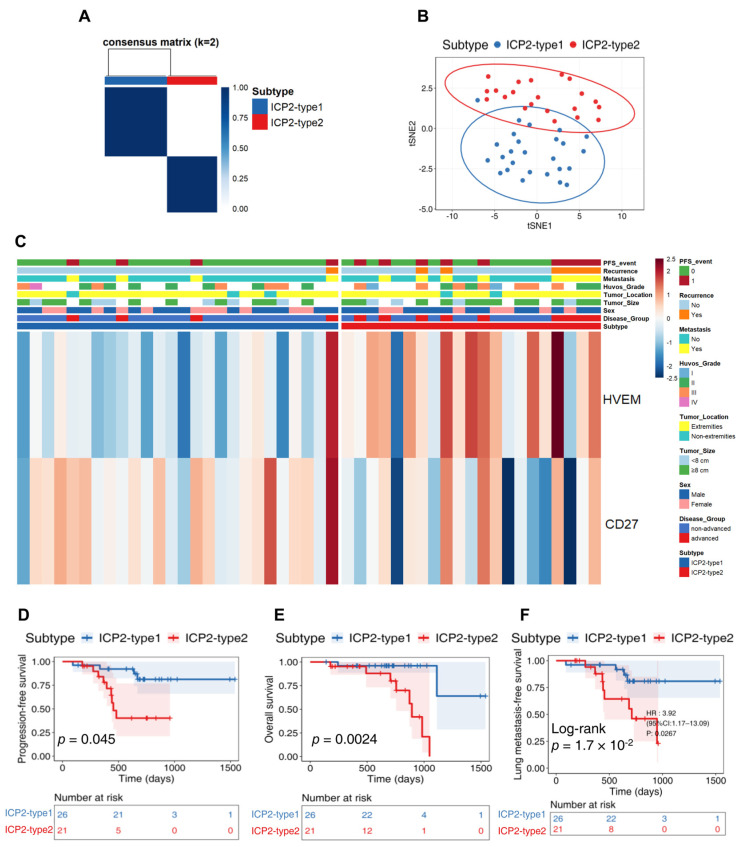
Consensus clustering of circulating ICPs identifies two prognostically distinct subtypes in osteosarcoma. (**A**) Consensus matrix heatmap from unsupervised clustering of sHVEM and sCD27 levels, showing stable partitioning into two clusters. (**B**) t-SNE projection providing a complementary visualization of the clustering structure. (**C**) Annotated heatmap showing z-scored sHVEM and sCD27 expression across patients, with clinical annotations including disease group, metastasis, recurrence, and other clinicopathologic variables. (**D**–**F**) Kaplan–Meier curves stratified by ICP2 subtype for PFS (**D**), OS (**E**), and lung metastasis-free survival (**F**). ICP2-type2 is associated with poorer outcomes across all endpoints.

Based on the circulating levels of sHVEM and sCD27, unsupervised consensus clustering classified the 47 patients with osteosarcoma into two groups (ICP2-type1, *n* = 26; ICP2-type2, *n* = 21). The consensus matrix showed stable partitioning into two clusters (Figure 2A). Additionally, t-SNE provided a complementary visualization supporting the clustering structure (Figure 2B).

ICP2-type2 was characterized by higher levels of both sHVEM and sCD27 and was associated with a higher frequency of adverse clinical features, including advanced disease status, metastasis during treatment, and recurrence (Figure 2C). Prior to clustering, sHVEM and sCD27 were selected from the full ICP panel through a three-step approach applied to the discovery cohort: (i) differential expression analysis comparing advanced versus non-advanced disease groups, (ii) univariable association with progression-free survival, and (iii) biological plausibility. Both markers demonstrated nominally significant differential expression and consistent survival associations and were therefore prioritized to construct the two-marker ICP2 signature.

Kaplan-Meier analysis demonstrated that patients in the ICP2-type2 group had significantly shorter PFS (log-rank *p* = 0.0045) and OS (log-rank *p* = 0.0024) compared to ICP2-type1 (Figure 2D,E). The lung metastasis-free survival was significantly reduced in the ICP2-type2 group (Figure 2F), Log-rank *p* < 0.001.

Multivariate Cox regression analyses, using Firth’s penalized likelihood to account for separation, confirmed that the ICP2 subtype was independently associated with both PFS and OS after adjusting for clinicopathological variables. Consistent results were observed in sensitivity analyses restricted to patients with complete tumor size data. The ICP2 model demonstrated strong discriminative performance, with apparent C-index values of 0.924 for PFS and 0.903 for OS (both exceeding 0.90; *n* = 32, events = 8 and 7, respectively) (Table S3). To assess optimism in this single-centre discovery cohort, internal validation was performed using LOOCV, in which consensus clustering of sHVEM and sCD27 was re-derived at each fold on the n − 1 training patients before Cox model fitting. LOOCV-corrected C-indices were 0.612 for PFS and 0.806 for OS. These corrected estimates are reported relative to the mean within-fold apparent C-indices of 0.687 for PFS (Δ = −0.075) and 0.689 for OS (Δ = +0.117), which are lower than the full-cohort apparent values of 0.924 and 0.903 because each LOOCV fold fits the model on only n − 1 patients, yielding a more conservative apparent performance. Taken together, these results confirm acceptable-to-good discrimination after accounting for overfitting (Figure S1).

### 3.4. Soluble Bone Remodeling Factors Are Not Differentially Expressed Between Disease Groups but Show Associations with Survival Outcomes

**Figure 3:**
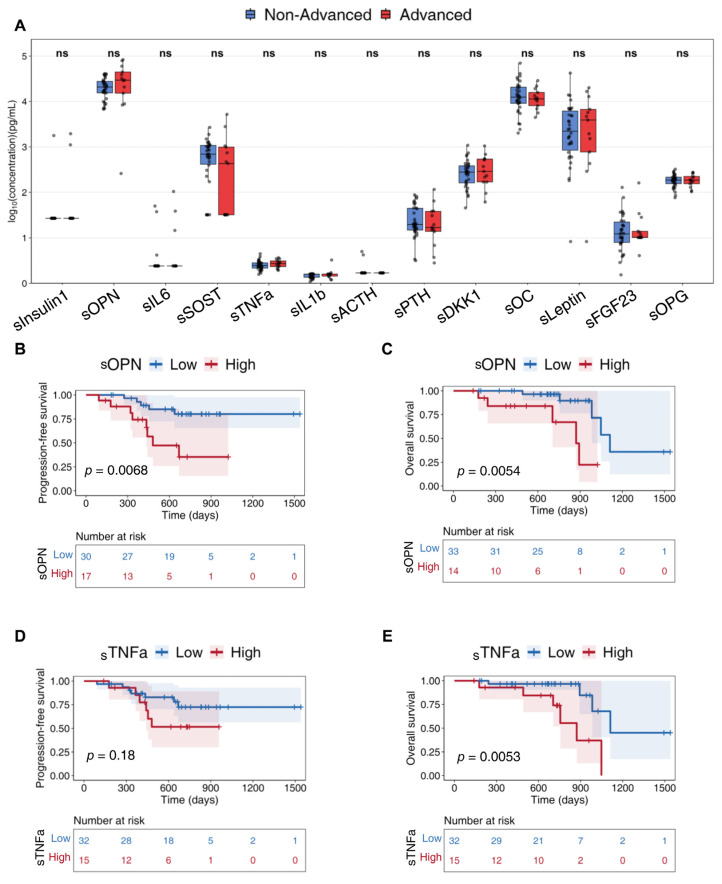
Soluble bone remodeling factors show limited group differences but prognostic associations in osteosarcoma. (**A**) Box plots showing circulating concentrations of 13 soluble BRFs (log10 pg/mL) in non-advanced (blue) and advanced (red) patients. Individual data points are overlaid. *p*-values were calculated using the Mann–Whitney U test with Benjamini–Hochberg correction; no significant differences were observed. (**B**,**C**) Kaplan–Meier curves for PFS (**B**) and OS (**C**) stratified by sOPN levels (Low, *n* = 30/33; High, *n* = 17/14 for PFS/OS, respectively). Elevated sOPN levels were associated with poorer outcomes. (**D**,**E**) Kaplan–Meier curves for PFS (**D**) and OS (**E**) stratified by sTNF-α levels (Low, *n* = 32; High, *n* = 15). sTNF-α shows no significant association with PFS but is associated with worse OS.

Circulating levels of 13 soluble BRFs were measured in plasma samples from patients with osteosarcoma. None of the analytes showed significant differences between the non-advanced and advanced groups after multiple test corrections (all adjusted *p* > 0.05; Figure 3A).

Next, we evaluated the prognostic relevance of individual markers using Kaplan–Meier analysis and Cox regression. These analyses were exploratory, with dichotomization based on data-driven cutoff points derived from the maximally selected rank statistics.

Among all markers, elevated sOPN levels were associated with shorter PFS (log-rank *p* = 0.0068; Figure 3B) and OS (log-rank *p* = 0.0054; Figure 3C). In univariate Cox analysis, high sOPN levels were associated with an increased risk of progression and death. After multivariate adjustment, the association with OS remained statistically significant, whereas the association with PFS was attenuated.

High sTNF-α levels were associated with worse OS (*p* < 0.001) in univariate analysis but showed no significant association with PFS (Figure 3D,E).

Given its consistent association with survival outcomes and its established role in bone remodeling and tumor progression, sOPN was selected as a representative bone remodeling factor for subsequent integrative analyses.

### 3.5. Integration of Immune Checkpoint and Bone Remodeling Biomarkers Contributes to Risk Stratification in Osteosarcoma

To evaluate whether combining the ICP2 subtype with the bone remodeling marker sOPN refines the prognostic stratification, we constructed an integrated classification model.

**Figure 4:**
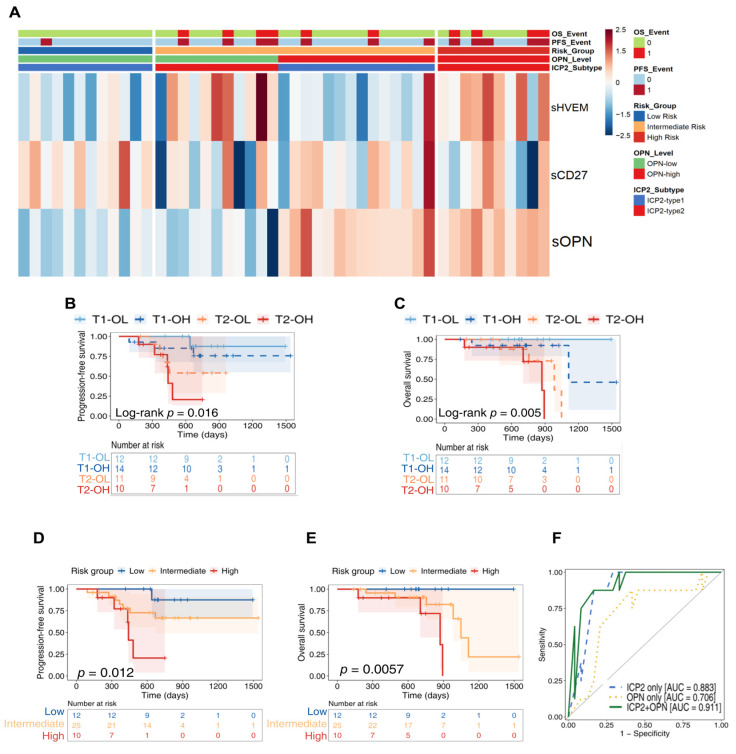
Integration of circulating immune checkpoint proteins and soluble osteopontin enables refined risk stratification in osteosarcoma. (**A**) Heatmap of z-scored log_2_-transformed concentrations (pg/mL + 1) of sHVEM, sCD27, and sOPN. Columns represent individual patients ordered from low to intermediate to high risk. Annotation bars (top to bottom): OS event (green = 0, red = 1), PFS event (light blue = 0, dark red = 1), risk group (blue/orange/red), sOPN level (green = low, red = high), and ICP2 subtype (blue = type1, red = type2). Color scale: red = higher expression, blue = lower expression (z-score capped at ±2.5). (**B**–**C**) Kaplan-Meier curves for PFS (**B**) and OS (**C**) across the four ICP2 × OPN subgroups. The ICP2-type2/OPN-high group showed the poorest outcomes. Log-rank *p*-values: PFS *p* = 0.016; OS *p* = 0.005. Numbers at risk are shown below each curve. (**D**) Kaplan-Meier curves for PFS stratified by integrated risk group (low, *n* = 12; intermediate, *n* = 25; high, *n* = 10). Log-rank *p* = 0.012. Median PFS was 441 days in the high-risk group and was not reached in the low- and intermediate-risk groups. (**E**) Kaplan–Meier curves for OS stratified by integrated risk group. Log-rank *p* = 0.0057. No OS events occurred in the low-risk group. Median OS was 1048 days in the intermediate-risk group and 872 days in the high-risk group. These estimates should be interpreted cautiously due to limited events. (**F**) ROC curves comparing logistic regression models for advanced disease status. Models include ICP2 alone (AUC = 0.883), sOPN alone (AUC = 0.706), and the combined ICP2 + OPN model (AUC = 0.911). Analysis was performed in patients with complete covariate data (*n* = 32). DeLong test *p*-values: ICP2 + OPN vs. ICP2 alone, *p* = 0.383; ICP2 OPN vs. OPN alone, *p* = 0.011.

Patients were categorized into four groups according to ICP2 subtype and sOPN level (ICP2-type1/OPN-low [T1-OL], ICP2-type1/OPN-high [T1-OH], ICP2-type2/OPN-low [T2-OL], and ICP2-type2/OPN-high [T2-OH]), which were further consolidated into a three-tier risk stratification scheme: low risk (ICP2-type1/OPN-low), intermediate risk (discordant groups, including ICP2-type1/OPN-high and ICP2-type2/OPN-low), and high risk (ICP2-type2/OPN-high) (Figure 4A).

Kaplan–Meier analysis across the four subgroups demonstrated a progressive separation of survival curves, with the T2-OH group exhibiting the poorest prognosis for both PFS (log-rank *p* = 0.016; Figure 4B) and OS (log-rank *p* = 0.005; Figure 4C). Notably, the discordant groups (T1-OH and T2-OL) showed intermediate survival patterns between the two extreme groups, supporting the rationale for subsequent risk consolidation.

The three-tier integrated risk classification demonstrated significant stratification of PFS (log-rank *p* = 0.012; Figure 4D). During follow-up, the median PFS was reached only in the high-risk group, whereas it was not reached in the low- and intermediate-risk groups, indicating a clear separation of risk categories. A similar trend was observed for OS (log-rank *p* = 0.0057; Figure 4E); however, these findings should be interpreted with caution due to the limited number of events, particularly in the low-risk group where no OS events were observed.

To assess incremental predictive performance, we compared logistic regression models based on ICP2 alone, sOPN alone, and the combined ICP2 + OPN model for discrimination of advanced disease status (Figure 4F). ICP2 alone showed strong discriminative performance (AUC = 0.883), while sOPN alone demonstrated moderate performance (AUC = 0.706). The combined model achieved the highest AUC (0.911); however, the improvement over ICP2 alone was not statistically significant (DeLong test, *p* = 0.383), indicating that the addition of sOPN did not substantially enhance overall discrimination in this setting. In contrast, the combined model performed significantly better than sOPN alone (*p* = 0.011).

Taken together, these results suggest that sOPN provides complementary information to ICP2, primarily by refining risk stratification within ICP2-defined subgroups rather than by markedly improving global discriminative performance. The apparent discrepancy between survival-based stratification and ROC analysis likely reflects differences in endpoint definition, as time-to-event outcomes capture longitudinal risk dynamics that are not fully represented by binary classification of advanced disease status.

## 4. Discussion

Osteosarcoma arises within a complex bone microenvironment in which tumor cells dynamically interact with immune cells, osteoblasts, osteoclasts, and stromal components. These interactions generate systemic signals that may be detectable in the peripheral blood. In this study, we demonstrated that a minimal circulating biomarker signature composed of two soluble ICPs (HVEM and CD27) and one BRF (OPN) could stratify patients with osteosarcoma into distinct prognostic groups using the earliest available plasma samples prior to disease progression.

Our results highlighted the central role of the immune microenvironment in osteosarcoma progression. HVEM is a member of the TNF receptor superfamily that functions as a bidirectional immune regulator capable of delivering either co-stimulatory or inhibitory signals, depending on ligand interactions [25,26]. HVEM signaling influences T-cell activation, NK-cell function, and immune tolerance in the tumor microenvironment [14]. Elevated circulating HVEM has been associated with poor outcomes in several hematological and solid malignancies [27,28,29]. In the present study, high plasma HVEM levels were strongly associated with inferior progression-free and OS, suggesting that systemic HVEM signaling may reflect an immunosuppressive tumor milieu in osteosarcoma.

CD27 is a costimulatory receptor expressed on T cells and memory B cells and plays an essential role in T-cell expansion and survival [30]. Although CD27 signaling typically promotes anti-tumor immunity, elevated soluble CD27 may indicate chronic immune activation or dysregulated T-cell signaling [31,32]. The combined elevation of circulating sHVEM and sCD27 observed in the ICP2-type2 subtype likely reflects a state of immune exhaustion or ineffective immune surveillance in aggressive osteosarcoma. By capturing two opposing poles of the adaptive immune balance, this two-marker panel provided robust exploratory prognostic value. Furthermore, because the majority of plasma samples were collected after completion of neoadjuvant chemotherapy, the observed circulating ICP profiles may partially reflect treatment-associated systemic immune modulation. Chemotherapy is known to induce immunogenic cell death and remodel the tumor immune microenvironment, suggesting that the ICP2 signature more likely reflects a dynamic, treatment-modulated immune state rather than a purely treatment-naïve baseline immune characteristic [33,34].

Crucially, the small size of our discovery cohort raises valid concerns regarding optimism bias and overfitting in predictive modeling [35]. To rigorously address this, we subjected our models to internal validation using LOOCV approach [36]. Reassuringly, the LOOCV-corrected C-indices demonstrated that the ICP2 panel maintains acceptable-to-good discriminative capacity-particularly for OS (LOOCV-corrected C-index = 0.806). This internal validation confirms that the prognostic stratification achieved by the HVEM/CD27 signature is driven by genuine biological signals rather than being purely a statistical artifact of the small sample size. Nevertheless, while LOOCV robustly penalizes for optimism, it cannot fully substitute for external testing, reinforcing the need to validate this signature in independent patient cohorts [37].

In contrast to ICPs, BRFs primarily reflect tumor–bone interactions. Osteosarcoma develops in a highly dynamic bone remodeling environment characterized by abnormal osteoclast activation and matrix turnover. sOPN is a multifunctional secreted glycoprotein involved in osteoclast recruitment, extracellular matrix remodeling, and immune modulation [38,39,40]. sOPN has been implicated in tumor invasion and metastatic dissemination in multiple cancer types [41,42].

Although the addition of sOPN showed only a modest, incremental improvement in statistical performance compared to the ICP2 panel alone, its inclusion provides a biologically coherent framework for risk stratification. OPN, also known as SPP1, serves as a multifaceted driver of tumor growth, metastasis, and chemoresistance in osteosarcoma. Specifically, OPN stimulates robust tumor cell proliferation by activating the PI3K/Akt signaling pathway and upregulating CCND1 expression, while simultaneously enabling cells to evade apoptosis and contributing to resistance against chemotherapy, such as doxorubicin [39,43,44]. Beyond the primary site, OPN facilitates the metastatic cascade by enhancing cell adhesion to the endothelium and activating matrix metalloproteinases (MMPs) to degrade the basement membrane, alongside promoting angiogenesis and metabolic reprogramming via GLUT1 and GLUT3 upregulation [38,43].

To minimize selection bias, we employed a pre-specified median cutoff for OPN. This exploratory integration defined a high-risk subgroup (ICP2-type2/OPN-high) with uniformly poor clinical outcomes and allowed refined stratification of an intermediate-risk subgroup where immune and bone-remodeling signals were discordant. This integrated signature represents a potential circulating indicator of the “immune–bone axis,” which drives osteosarcoma progression. Moreover, the biomarker panel required only three analytes that were measurable in the plasma, suggesting its potential feasibility for clinical translation.

While this study provides proof-of-concept evidence for a circulating biomarker-based risk stratification approach in osteosarcoma, several limitations should be acknowledged. First, the cohort size was relatively small (*n* = 47) and derived from a single center leading to a marginal Events Per Variable ratio in our multivariable models. This inherently produced “step-like” Kaplan–Meier survival curves with wide confidence intervals and partial separation in multivariable models (addressed via Firth’s penalized likelihood approach). Consequently, the high apparent C-index of our models carries a risk of overfitting and requires further validation in a larger independent cohort.

Second, the presence of missing clinical data for variables such as tumor size, ALP, and LDH-which is common in prospective cohorts of rare malignancies-may have limited our ability to fully adjust for all potential confounders in the multivariable analyses. Nevertheless, the prognostic performance of the ICP2 signature was consistent across both the full cohort and the complete-case sensitivity analyses, supporting the robustness of our conclusions.

Third, plasma samples were collected at two pre-specified time points: baseline prior to neoadjuvant chemotherapy (*n* = 12) and immediately before surgery following completion of neoadjuvant chemotherapy (pre-surgery; *n* = 35). Consequently, circulating ICP levels measured after neoadjuvant chemotherapy may differ from treatment-naïve baseline states, potentially confounding multivariable analyses. However, these post-chemotherapy samples may also reflect clinically relevant treatment-modulated tumor–immune interactions and residual disease biology in osteosarcoma.

Fourth, sHVEM and sCD27 were selected based on both statistical significance and strong biological rationale but without formal multiple-testing correction; therefore, the resulting ICP2 panel remains hypothesis-generating and requires validation in a larger, independent cohort to confirm its generalizability.

Despite these limitations, the present study provides proof-of-concept evidence that circulating ICPs and BRFs jointly capture the biologically relevant aspects of osteosarcoma progression. Future studies integrating circulating biomarkers with tumor transcriptomics, single-cell analyses, and spatial profiling may further clarify the immune–bone interactions that shape osteosarcoma evolution.

## 5. Conclusions

In conclusion, this study demonstrates that a minimally invasive liquid biopsy integrating immune and bone-remodeling signals can effectively refine risk stratification in osteosarcoma. Specifically, a two-marker panel of circulating sHVEM and sCD27 (ICP2) serves as a hypothesis-generating indicator of the systemic immune state, successfully stratifying patients into distinct prognostic subtypes. Furthermore, the exploratory addition of OPN provides biologically relevant supplementary value by identifying an intermediate-risk subgroup driven by tumor–bone interactions. Upon external validation, this simple three-analyte profiling approach has the potential to complement existing clinical staging systems, thereby facilitating personalized monitoring and treatment strategies for osteosarcoma patients.

## Figures and Tables

**Table 1:** Clinical and demographic characteristics of non-advanced and advanced osteosarcoma patients.

Variable	Total (*N* = 47)	Non-Advanced (*N* = 34)	Advanced (*N* = 13)	*p*-Value
**Demographics**				
Age	16.0 [12.0, 18.0]	15.5 [12.2, 18.0]	16.0 [12.0, 18.0]	0.774
BMI ^a^	18.1 [16.4, 20.1]	17.6 [16.4, 19.3]	19.0 [17.1, 21.1]	0.301
Sex: Male	29 (61.7)	20 (58.8)	9 (69.2)	0.748
**Tumor Characteristics**				
Tumor location:				
Non-extremities	5 (10.6)	3 (8.8)	2 (15.4)	0.902
Tumor size(cm) ^b^	10.9 [7.0, 14.0]	9.8 [7.0, 13.2]	12.3 [10.9, 14.1]	0.231
Tumor size ≥ 8 cm ^b^	22 (68.8)	15 (62.5)	7 (87.5)	0.378
Histological subtype				0.195
Osteoblastic	44 (93.6)	33 (97.1)	11 (84.6)	
Chondroblastic	2 (4.3)	1 (2.9)	1 (7.7)	
Telangiectatic	1 (2.1)	0 (0.0)	1 (7.7)	
Enneking stage				1.000
Stage IB	2 (4.3)	2 (5.9)	0 (0.0)	
Stage IIB	45 (95.7)	32 (94.1)	13 (100)	
Stage III	0 (0.0)	0 (0.0)	0 (0.0)	
**Laboratory Values**				
ALP(U/L) ^c^	121.0 [94.2, 196.6]	106.4 [84.8, 173.3]	194.9 [115.5, 253.5]	0.022
LDH(U/L) ^c^	188.0 [163.5, 240.8]	183.0 [168.0, 234.0]	205.0 [159.0, 283.0]	0.936
**Treatment Response**				
Huvos grade ^d^				0.716
Grade I	2 (7.7)	2 (10.5)	0 (0.0)	
Grade II	11 (42.3)	8 (42.1)	3 (42.9)	
Grade III	12 (46.2)	8 (42.1)	4 (57.1)	
Grade IV	1 (3.8)	1 (5.3)	0 (0.0)	
Huvos good response (≥90% necrosis) ^d^	13 (50.0)	9 (47.4)	4 (57.1)	1.000
**Disease Events and Outcomes**				
Metastasis at diagnosis	0 (0.0)	0 (0.0)	0 (0.0)	NA
Metastasis during treatment	12 (25.5)	0 (0.0)	12 (92.3)	<0.001
Recurrence	7 (14.9)	0 (0.0)	7 (53.8)	<0.001
OS event (death)	10 (21.3)	0 (0.0)	10 (76.9)	<0.001
PFS event	13 (27.7)	0 (0.0)	13 (100)	<0.001
PFS (days)	617.0 [384.5, 742.0]	680.0 [433.0, 833.0]	395.0 [320.0, 454.0]	0.003
OS, (days)	693.0 [463.5, 868.0]	Not reached	711.0 [518.0, 893.0]	0.461

Data are shown as median [IQR] or number (%). Abbreviations: ALP = alkaline phosphatase; BMI = body mass index; IQR = interquartile range; LDH = lactate dehydrogenase; NA = not applicable; OS = overall survival; PFS = progression-free survival. *p*-values from Mann–Whitney U test (continuous variables) or Fisher’s exact test/Pearson’s χ^2^ (categorical variables). ^a^ BMI missing for 8 patients (17.0% missing overall). ^b^ Tumor size missing for 15 patients (31.9% missing overall); categorical analysis based on available data. ^c^ ALP and LDH are missing for 19 patients (40.4% and 44.7% missing overall, respectively). ^d^ Huvos grade available in 26 evaluable patients (44.7% missing overall); good response defined as ≥90% necrosis.

## Data Availability

The data presented in this study are available from the corresponding author upon reasonable request. Access to the data is subject to institutional approval and compliance with ethical and privacy regulations.

## Supplementary Materials

The following supporting information can be downloaded at: https://www.mdpi.com/article/10.3390/cancers18111701/s1, Figure S1. Leave-one-out cross-validation (LOOCV) assessment of ICP2 model discrimination; Table S1: Differential expression of immune checkpoint proteins between groups; Table S2: Logistic regression analysis of individual soluble immune checkpoint protein analytes for predicting advanced osteosarcoma (*n* = 47); Table S3: Penalized Cox regression and model performance statistics for ICP2 and BRF1 panels in survival analysis.

## Abbreviations

The following abbreviations are used in this manuscript:

ACTH	Adrenocorticotropic hormone
ALP	Alkaline phosphatase
BMI	Body mass index
BRF	Bone remodeling factor
BTLA	B and T lymphocyte attenuator
CD27	Cluster of Differentiation 27
CD28	Cluster of Differentiation 28
CD40	Cluster of Differentiation 40
CD80 (B7-1)	Cluster of Differentiation 80
CD86 (B7-2)	Cluster of Differentiation 86
CTLA-4	Cytotoxic T-lymphocyte-associated protein 4
DKK-1	Dickkopf-related protein 1
EDTA	Ethylenediaminetetraacetic acid
FGF-23	Fibroblast growth factor 23
GITR	Glucocorticoid-induced TNFR-related protein
GITRL	GITR ligand
HVEM	Herpesvirus Entry Mediator (TNFRSF14)
ICOS	Inducible T-cell co-stimulator
ICPs	Immune checkpoint proteins
ICP2	Two-marker ICP signature
IL-1β	Interleukin-1 beta
IL-6	Interleukin-6
CI	Confidence Interval
IQR	Interquartile ranges
LAG-3	Lymphocyte activation gene-3
LDH	Lactate dehydrogenase
LOOCV	Leave-one-out cross-validation
MCAR	Missing Completely at Random
MinDC	Minimum detectable concentrations
MMPs	Matrix metalloproteinases
NA	Not applicable
OPN	Osteopontin
OS	Overall survival
PCA	Principal Component Analysis
PD-1	Programmed cell death protein 1
PD-L1	Programmed death-ligand 1
PD-L2	Programmed death-ligand 2
PFS	Progression free survival
ROC	Receiver operating characteristic
SA-PE	Streptavidin-phycoerythrin
TIM-3	T-cell immunoglobulin and mucin-domain containing-3
TLR-2	Toll-like receptor 2
TNF-α	Tumor necrosis factor alpha
t-SNE	t-Distributed Stochastic Neighbor Embedding
