# Supplementary figures and images for: Integration of Circulating Immune Checkpoint Proteins and Osteopontin Refined Risk Stratification in Osteosarcoma
Source: Cancers (Basel). 2026 May 23;18(11):1701. doi: 10.3390/cancers18111701 (PMC13255615; doi:10.3390/cancers18111701)

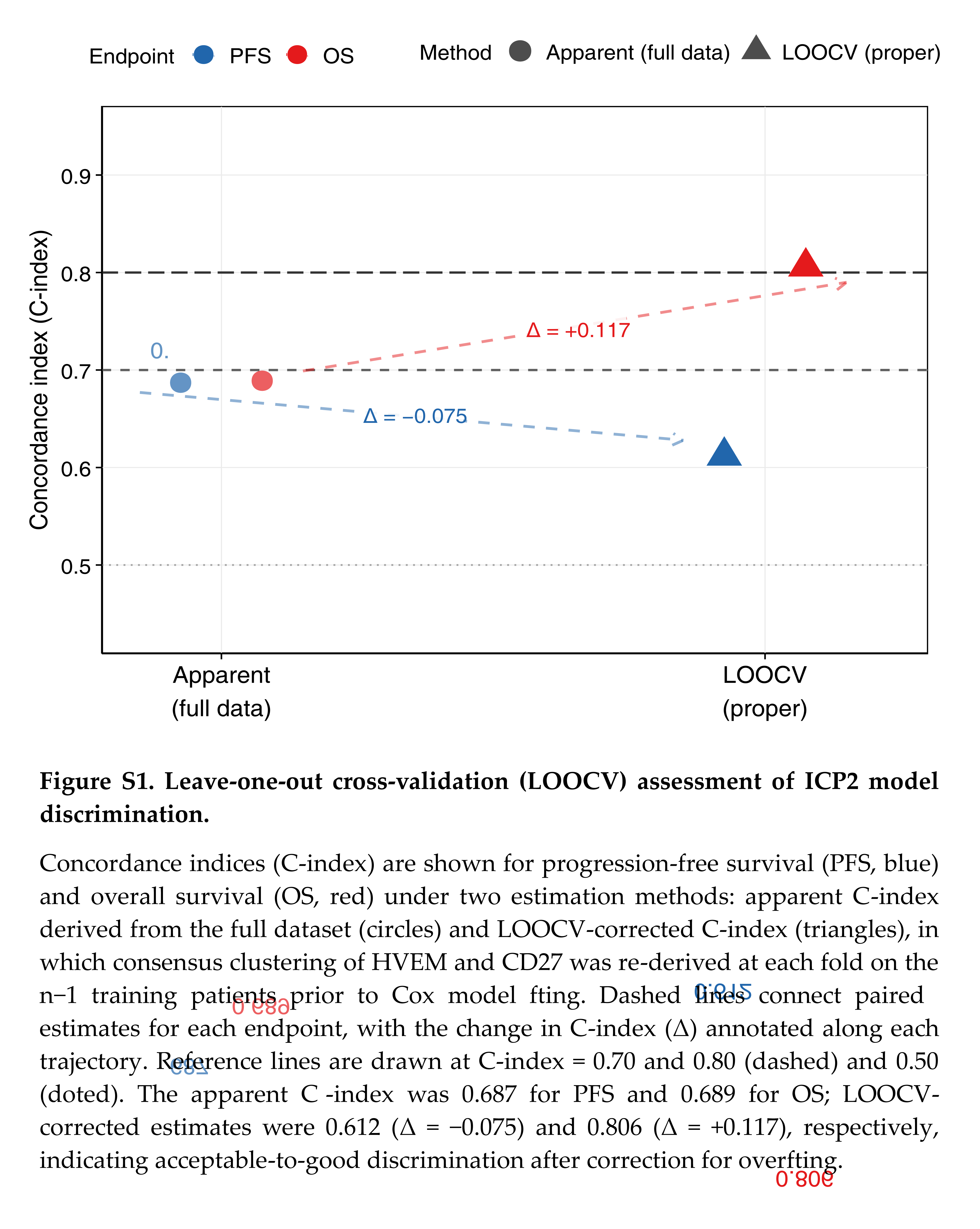

Supplement: Supplementary file 1 [file cancers-18-01701-s001.zip › Figure S1.tif]
